# The Priming Effects of Mirror Visual Feedback on Bilateral Task Practice: A Randomized Controlled Study

**DOI:** 10.1155/2019/3180306

**Published:** 2019-11-26

**Authors:** Yi-chun Li, Ching-yi Wu, Yu-wei Hsieh, Keh-chung Lin, Grace Yao, Chia-ling Chen, Ya-Yun Lee

**Affiliations:** ^1^School of Occupational Therapy, College of Medicine, National Taiwan University, Taipei, Taiwan; ^2^Department of Occupational Therapy and Graduate Institute of Behavioral Sciences, College of Medicine, Chang Gung University, Taoyuan, Taiwan; ^3^Healthy Aging Research Center, Chang Gung University, Taoyuan, Taiwan; ^4^Department of Physical Medicine and Rehabilitation, Chang Gung Memorial Hospital, Linkou, Taiwan; ^5^Division of Occupational Therapy, Department of Physical Medicine and Rehabilitation, National Taiwan University Hospital, Taipei, Taiwan; ^6^Department of Psychology, National Taiwan University, Taipei, Taiwan; ^7^Graduate Institute of Early Intervention, College of Medicine, Chang Gung University, Taoyuan, Taiwan; ^8^School and Graduate Institute of Physical Therapy, College of Medicine, National Taiwan University, Taipei, Taiwan

## Abstract

The priming effect of mirror visual feedback can be simply provided by inexpensive mirror therapy (MT), which exhibits beneficial effects on sensorimotor recovery in stroke. The present study was a single-blind pretest-posttest study that examined whether the priming effect of mirror visual feedback on bilateral task practice would render better outcomes. Twenty-three patients with chronic stroke were randomized to receive hospital-based task-oriented MT or bilateral arm training (BAT) for 4 weeks at 90 minutes/day, 3 days/week and a home practice for 30-40 minutes/day, 5 days/week. There was the potential trend for MT to improve temperature sense as measured by the revised Nottingham Sensory Assessment (Cohen's *d* = 1.00; 95% confidence interval, -0.09 to 2.09), and MT increased the Stroke Impact Scale 3.0 total score (*d* = 0.89; 0.003 to 1.71). MT also showed a trend for greater improvements in the Motor Activity Log (amount of use: *d* = 0.62; -0.24 to 1.44; quality of movement: *d* = 0.50; -0.35 to 1.31). MT involving bilateral movement practice with the priming effect of mirror visual feedback may render beneficial effects. The unilateral approach or MT augmented by extra feedback might be appropriate modifications.

## 1. Introduction

Stroke is a leading cause of disability worldwide. Stroke survivors may have impairments in sensorimotor function [1–3]. Activities of daily living and quality of life are thus negatively affected. Priming is a type of implicit learning that can increase the excitability of the cortex and optimize rehabilitation outcomes [4], and active treatments can induce priming effects.

Among a wide range of stroke interventions, mirror therapy (MT) and bilateral arm training (BAT) are novel therapies for stroke. These interventions are priming techniques based on the bilateral approach to stroke rehabilitation and are easy for implementation during occupational therapy [4–6]. During MT, patients are provided with visual feedback of normal movement of the unaffected arm from the mirror [7]. Based on findings from functional neuroimaging or electrophysiological techniques, the interhemispheric imbalance caused by stroke may be revised by mirror visual feedback [8]. The effects of MT on motor function and activities of daily life were also shown using standard meta-analysis [9, 10]. With repetitive, bilateral, and symmetrical movement practice, different MT protocols for stroke rehabilitation have been developed, including task-oriented and non-task-oriented practices [11, 12]. These protocols have further shown that MT is effective in improving sensorimotor performance and activities of daily living and that these findings are explained by the possible mechanisms of increasing cognitive penetration in action control, activation of the mirror neuron system after training, and the modulatory effects on motor and sensory networks [5, 11–14]. A previous study showed that engaging in activities of daily living is associated with a better quality of life in individuals with chronic stroke [15].

BAT has been provided in different forms, such as symmetric or alternating patterns, in task-oriented or non-task-oriented practice, and BAT has been implemented with electromyography-triggered neuromuscular stimulation, robots, or auditory cueing [16]. The effect of BAT is speculated to result from the recruitment of ipsilateral corticospinal pathways, increasing control from the contralesional hemisphere, and normalization of inhibitory mechanisms [6]. A previous study that concentrated on symmetric and task-oriented protocols demonstrated that BAT is effective in improving motor control and motor function of the affected arm in stroke patients [17].

MT and BAT share similar key therapeutic elements, including the use of simultaneous bilateral arm movements, mass, and repetitive practice and providing movement-based priming. Conversely, the difference between BAT and MT lies in the mirror visual feedback in MT, which involves perceptual incongruence between visual and somatosensory areas and may offer a priming effect on motor learning [4, 5].

In the past, MT was used as a priming technique to improve affected arm function and occupational performance for stroke [18, 19]. Numerous studies have investigated the effects of MT compared with/without BAT; however, the priming effect of mirror visual feedback on bilateral task practice was not clearly examined in rigorous studies [12, 13, 20–23]. Previous research showed that task-oriented MT combined with BAT was effective in improving the sensorimotor performance of the affected arm [12, 13]. However, participants in the control group underwent conventional therapy, and whether the mirror visual feedback in MT was the pivotal element of the bilateral task practice was not clear. Four studies have compared the effects of MT with those of BAT in chronic stroke patients, but the results are questionable [22, 23]. In Antoniotti et al.'s study [20], the control group received sham therapy in which an opaque surface replaced the mirror/reflecting surface, which may be less powerful than BAT. The other training programs in two studies were conducted in the home settings of the patients. The difficulty of monitoring the dose of in-home training might be a factor that decreases the possible efficacy of that treatment program [22, 23]. Furthermore, the outcome measures used in the studies [21–23] relied on motor performance and were limited in revealing specific changes in sensory recovery, which is necessary for revealing the mechanisms underlying interventional approaches and is beneficial to functional recovery for the more than 60% of stroke patients who manifest sensory deficits [24].

This study evaluated the priming effects of mirror visual feedback by comparing the effects of task-oriented MT and BAT on sensorimotor performance and quality of life among chronic stroke patients who received an equal amount of therapy. We hypothesized that bilateral task practice with visual mirror feedback would lead to greater improvement in the outcome measures than bilateral task practice alone.

## 2. Materials and Methods

### 2.1. Participants

**Figure 1:**
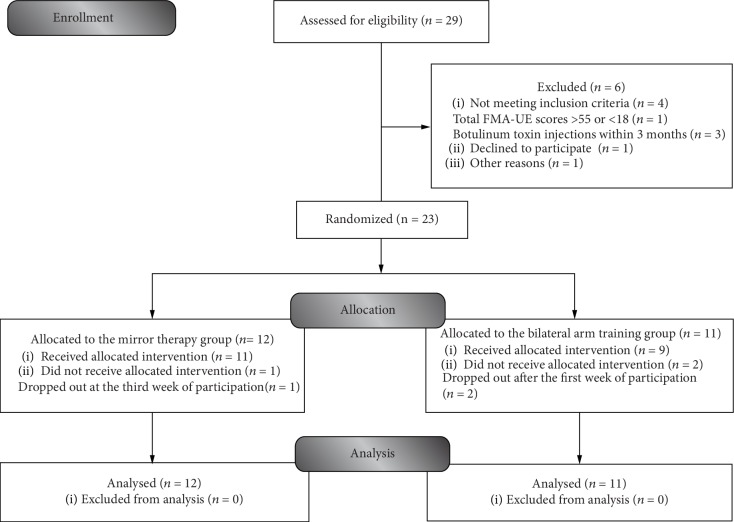
Flow diagram of participants in the study.

Individuals were recruited from 4 participating sites, including 1 medical center and 3 regional hospitals, upon the institutional review board approval. Using hospital records, once the potential participants were identified by the study therapist, the participants were invited and explained the experimental procedures of this study. Further eligibility and baseline assessments were then undertaken by the study assessor. The diagnosis of stroke was performed using standard imaging techniques. The inclusion criteria were that the patient had sustained their first-ever unilateral ischemic or hemorrhagic stroke more than 6 months after the onset; had mild to moderate motor impairment (total Fugl-Meyer Assessment upper extremity score between 18 and 55) [7, 25]; had no severe spasticity in any joint of the affected arm (modified Ashworth Scale score < 3) [26]; was able to follow instructions; had no serious vision deficits (based on the best gaze score on the National Institutes of Health Stroke Scale) [27]; had no other neurologic, neuromuscular, or orthopedic disease; was not simultaneously participating in other studies; and had not received botulinum toxin injections within the previous 3 months. This study was registered at ClinicalTrials.gov, was in accordance with the ethical guidelines of the revised (2000) Helsinki Declaration, and was approved by the Human Research Committee. Informed consent and assent, which included the study's risks and benefits, were obtained orally and in writing from each participant. All participation was voluntary and anonymous. Patient confidentiality and data security were appropriately handled. To date, no published research has compared the effects of bilateral task practice with visual mirror feedback with those of bilateral task practice alone on sensorimotor performance and quality of life among chronic stroke patients who received an equal amount of therapy. Thus, the sample size required for this project was calculated and estimated based on previous studies [12, 13]. Based on the smallest sample size needed for achieving a statistical power of 0.80 with a one-sided type I error of 0.05, a total sample size of at least 11 subjects per group was deemed sufficient. More information on enrollment is shown in Figure 1.

### 2.2. Design

The study used a single-blind randomized pretest and posttest design. Based on a computer-generated random-sequence table, permuted-block randomization to the BAT or MT groups (Figure 1) was performed by an independent research assistant, and the participants were stratified by the lesion side (right or left) and total Fugl-Meyer Assessment upper extremity pretest scores (<40 or ≥40) [7]. The allocation ratio was 1 : 1 for the two groups. Interventions and outcome assessments were administered, respectively, by two well-trained and certified occupational therapists. Therapy and measurement supervision were provided by scheduled meetings, communication software, telephone, and record sheets between therapists and investigators. The assessor responsible for the outcome measure was blinded to the group assignments of the participants, who were blinded to the study hypotheses. Outcome measures were administered to participants at the baseline and immediately after the intervention.

### 2.3. Interventions

The treatment regimens were designed so that both groups received an equal amount of therapy, which included 4 weeks with (1) 1.5 hours/day, 3 days/week of hospital-based MT or the BAT protocol and (2) 30 to 40 minutes/day, 5 days/week of home practice. The hospital-based therapy was conducted during the participants' regularly scheduled occupational therapy sessions. All other routine interdisciplinary stroke rehabilitation methods were continued as usual throughout the study.

#### 2.3.1. Hospital-Based MT Protocol

**Figure 2:**
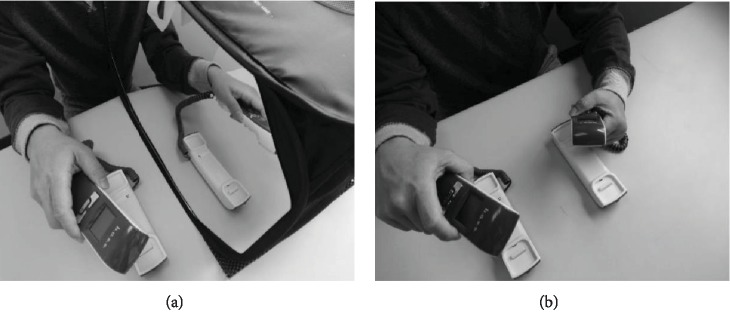
Intervention setup for mirror therapy (a) and bilateral arm training (b).

The hospital-based MT protocol included mirror box training for 45 minutes and functional training for 45 minutes. After 10 minutes of warm-up exercises for the affected arm, including stretching and a passive range of motion exercises, a portable mirror box (48 × 36 × 36 cm^3^) [28] was placed in the midsagittal plane of each participant. The affected arm was positioned behind the mirror. The movements of the unaffected arm in front of the mirror were reflected as if the affected side was being moved (Figure 2(a)). During the mirror box training, the participants were guided to gaze at the mirrored image to allow them to imagine that the reflection was their affected arm performing the activities and to move both arms in symmetric patterns as simultaneously as possible. The activities consisted of 10 minutes of non-task-oriented movements, such as forearm pronation/supination or finger flexion/extension, and 35 minutes of task-oriented activities, such as picking up the handset from the phone, picking up items and putting them in the box, or other functional tasks involved in daily activities.

MT was followed by 45 minutes of functional training, such as chopping vegetables and pouring water from a kettle. All movements and activities during the functional training were designed according to the impairments of the participants and their individual rehabilitation goals.

#### 2.3.2. Hospital-Based BAT Protocol

The hospital-based BAT protocol was similar to that of MT, but the mirror box was not provided (Figure 2(b)). The participants were asked to symmetrically move both arms as simultaneously as possible.

#### 2.3.3. Home Practice

Home practice was customized to each participant's regular environment to achieve the purpose of rehabilitation. The activities were selected, demonstrated, and repeatedly practiced throughout the functional training session in the hospital to confirm that the patient performed them correctly. To ensure completeness, we informed the participants about the decline in effect without practice and had them complete a form that included their name, procedure, repetition frequency, and duration of the activities and the problems they encountered while performing the activities. The therapist conducted follow-ups by telephone and communication software or at each hospital visit.

### 2.4. Outcome Measures

The outcome measures used in the study covered the International Classification of Functioning, Disability and Health (ICF) domains of body function, structure, activity, and participation and included the Fugl-Meyer Assessment, the revised Nottingham Sensory Assessment, the Chedoke Arm and Hand Activity Inventory, the Motor Activity Log, and the Stroke Impact Scale 3.0. The amount of therapy (the repetition frequency of 10 minutes of non-task-oriented movements plus 35 minutes of task-oriented activities) administered to the participants and potential adverse effects, including pain and fatigue, were also recorded during the intervention period.

#### 2.4.1. Primary Outcomes

The level of upper extremity motor impairment was evaluated with the 33-item Fugl-Meyer Assessment, which uses a 3-point scale (0 to 2) [25]. The interrater reliability and reproducibility of this instrument have been established [25]. The revised Nottingham Sensory Assessment was used to assess sensation impairments. A 3-point scale (0 to 2), with a total score of 48 points, assessed the tactile subtest, which included light touch, temperature, pinprick, pressure, tactile localization, and bilateral simultaneous touch on the shoulder, elbow, wrist, and hand. We analyzed the data of the participants who scored less than 48 points (indicating sensation impairment) at the pretest [12]. The reliability has been well established [29]. The Stroke Impact Scale 3.0 was used to evaluate self-perceived quality of life and multidimensional stroke recovery, including four physical functions, memory, emotion, communication, and social participation domains. The Stroke Impact Scale 3.0 is established on a 5-point scale (1 to 5). An overall mean total score was calculated [30]. This instrument was developed by Rasch analysis and has shown good validity [31].

#### 2.4.2. Secondary Outcomes

The Chedoke Arm and Hand Activity Inventory measures arm and hand functions on 13 real-life bilateral tasks on a 7-point scale (1 to 7) [32]. The properties of the Chedoke Arm and Hand Activity Inventory have been studied, showing high interrater reliability and convergent and discriminant cross-sectional validity [32]. The 30-item Motor Activity Log, a semistructured interview, evaluated self-perceived real-world use, including the amount of use and quality of movement of the affected upper extremity. The Motor Activity Log score with established reliability and validity was developed with a 6-point scale (0 to 5), from which the mean amount of use and quality of movement scores are calculated [33].

### 2.5. Statistical Analysis

Baseline clinical characteristics were presented as frequencies with percentages, means with standard deviations (SDs), or medians with the interquartile ranges. The differences between the two groups were compared by Fisher's exact test for categorical data and by the independent *t*-test or Mann–Whitney *U* test for continuous data, according to the distribution characteristics of the data [34–36]. To index the magnitude of the difference between the two groups, an effect size (Cohen's *d*) estimate and the 95% confidence interval (CI) were calculated [37, 38]. A large effect was represented by *d* of at least 0.8, a moderate effect by *d* of 0.5, and a small effect by *d* of 0.2. Improvements in each variable that exceeded 10% indicated clinically significant gains (minimal clinically important difference). The significance (*α*) level was set at 0.05 for all comparisons. The analyses were accomplished using SAS 9.4 software (SAS Institute Inc., Cary, NC) and G∗Power 3.1 [39]. Adverse effects were descriptively summarized.

## 3. Results

Twenty-three stroke patients (13 men and 10 women) consented to participate in the study. The patients had a mean age of 54.57 years (SD, 10.52 years; range, 41.16 years) with a mean stroke onset of 53 months (SD, 31.58 months) (Figure 1). The baseline characteristics of the participants in the two groups did not significantly differ (Table 1). No intolerable adverse effects were reported, and no significant difference was found in the average repetition frequency of 10 minutes of non-task-oriented movements and 35 minutes of task-oriented activities between the MT group (mean, 161.35 (SD, 34.24)) and the BAT group (mean, 168.10 (SD, 47.56); mean differences, -6.75 (95% CI, -40.89 to 27.39)).

### 3.1. Outcome Measures

#### 3.1.1. Primary Outcomes

No significant difference in the Fugl-Meyer Assessment was found between the two groups (Table 2). The MT group showed a potential trend for improved temperature sense as measured by the revised Nottingham Sensory Assessment (median difference, 0 (95% CI, 0.14 to 2.74); *d* = 1.00 (95% CI, -0.09 to 2.09)). Furthermore, four participants (44.44%) in the MT group achieved a minimal clinically important difference of ≥0.8 for the revised Nottingham Sensory Assessment temperature subtest. One participant (16.67%) in the BAT group achieved this standard, although this achievement failed to reach significance (*p* = 0.29 by Fisher's exact test). The MT group also demonstrated a significant and large improvement on the Stroke Impact Scale (mean differences, 5.82 (95% CI, 0.40 to 11.24); *d* = 0.89 (95% CI, 0.003 to 1.71)). The achievements of the minimal clinically important differences for the Motor Activity Log (amount of use and quality of movement) and the Stroke Impact Scale failed to reach significance between the two groups (*p* = 0.15, 0.28, and 0.12, respectively, by Fisher's exact test). According to the results, there was a trend for the MT group to improve in temperature sense and life quality as measured by the Stroke Impact Scale.

#### 3.1.2. Secondary Outcomes

No statistically significant difference in the Chedoke Arm and Hand Activity Inventory was found between the two groups (Table 2); however, clinically relevant improvements (effect size) in the Motor Activity Log scores of the MT group exceeded those of the BAT group (amount of use: mean differences, 0.30 (95% CI, -0.09 to 0.69); *d* = 0.62 (95% CI, -0.24 to 1.44); quality of movement: mean differences, 0.24 (95% CI, -0.14 to 0.62); *d* = 0.50 (95% CI, -0.35 to 1.31)). Summarizing these results, the MT group showed a trend for greater improvements in the Motor Activity Log scores than the BAT group.

## 4. Discussion

To the best of our knowledge, this study is the first to evaluate the priming effects of mirror visual feedback by comparing the effects of task-oriented MT and BAT on sensorimotor performance and quality of life among chronic stroke patients who received an equal amount of therapy. This study highlights the mirror visual feedback, which may be a pivotal element of bilateral task practice in MT, and provides different recovery characteristics. The findings partially support our hypothesis, with greater improvements in temperature sense and quality of life after MT.

### 4.1. Benefits of Mirror Visual Feedback

A previous study showed that MT has a large effect and demonstrated group differences in changes in temperature sense [12]. That study interpreted the promotion of temperature sense recovery as the result of the mirror visual feedback input that modulates the somatosensory cortex network via the mirror visual feedback to the multimodal neurons in the posterior parietal and premotor cortex. Furthermore, the recovery of temperature sense usually precedes the recovery of other somatosensory functions. The research design of the present study extends previous findings that recovery may be induced by the priming effect of mirror visual feedback. Neuroimaging techniques may be used to study neuroplastic changes in the brain corresponding to the above results.

In addition, although no statistically significant differences were found from before to after treatment in the outcome measures of motor impairment and arm/hand functions on bilateral tasks, the MT group showed significant improvements in quality of life and exhibited a trend for a greater extent of improved amount of use and quality of movement in daily life than the dose-matched BAT group. These results could be explained by an increased activation of the mirror neuron system and the priming effect of mirror visual feedback. The perceptual and motor areas may be connected by the mirror neuron system [5].

Furthermore, according to the priming paradigms, mirror visual feedback from MT provides priming effects not only by movement-based priming but also by further motor imagery and action observation [4]. These additional priming effects may also promote the participants' perception of strength and affected arm use, thereby improving the quality of life. To enhance the treatment effect during MT, increasing the weight of the manipulated objects or the speed of movements may be a direction for clinical practice.

### 4.2. No Significant Difference in the Improvement of Motor Impairment between Groups

The descriptive data showed that the improvement of the motor impairment and arm/hand functions on bilateral tasks was not significantly different between groups. Several possible reasons may explain these observations. First, a previous study showed bilateral reaching for targets that shifted from within to beyond the length of the arm increasing the recruitment of arm movements [40]. The size of the mirror box used in our study may have limited the movements of the participants in the MT group.

Second, the bilateral MT used in the study may have led to a limited deployment of attention to the mirror and practice of simultaneous bilateral movements for the participants with somatosensory impairment or poor attention. The possible efficacy might have also been decreased during bilateral MT.

### 4.3. Limitations and Future Directions

This preliminary study has several limitations that warrant consideration. First, this study was based on a small sample size. Further research based on a larger sample is needed to validate and extend the findings. For example, given a power of 0.80 and a one-sided type I error of 0.05, the minimum sample size for future trials to validate the advantages of MT in improving somatosensory function (e.g., temperature) will be at least 14 subjects in each intervention group. Second, we did not consider the effect of individualized target distance on the arm and trunk movement during reaching. This aspect is probably a factor contributing to the differences observed between these two groups and needs to be tested or well controlled in future studies.

Third, the bilateral MT used in the study may have also led to a limitation of deploying attention to the mirror and the practice of bilateral movements. The nature of mirror visual feedback experienced by the participants in the MT group may have differed on an individual basis and warrants scrutiny. The unilateral approach to MT or MT augmented by auditory feedback might be appropriate modifications in future research.

Finally, the demographic characteristics of the study participants should be considered when interpreting the findings of the study. Patients with hemorrhagic stroke may display better functional improvements over time [41]. However, the influence of stroke subtype on MT/BAT is undetermined. Our sample size was limited to allow a subgroup analysis. The potential influence of stroke subtype on the outcomes of MT and BAT awaits scrutiny based on a larger study.

Age may also be a moderator factor of the priming effect of mirror visual feedback in stroke that warrants evaluation. One study suggested that the activation of the mirror neuron system was independent of age [42]. However, the evidence for a direct relationship between age of onset of stroke and the priming effect of mirror visual feedback was insufficient. In light of Radajewska's study, older stroke patients may be more likely to experience the advantages of better functional outcomes after MT [43]. In our study, the sample size was considerably small to make a meaningful statistical analysis of the age effect on the outcomes of MT. Since age may influence the activation of the mirror neuron system, the relation of age to the priming effect of mirror visual feedback waits further scrutiny in future research.

Social or personal factors, such as family support or general health status, might influence treatment outcomes, which may further affect the performance of or participation in meaningful occupations [44, 45]. Future research may consider incorporating the abovementioned factors in a larger sample to validate and extend our findings. In addition, the outcome measures used in the present study are component based. In order to identify whether the desired outcomes have been achieved after intervention, future research may extend to use assessments of the performance of or participation in meaningful occupations, such as the Satisfaction with Performance Scaled Questionnaire (SPSQ) [1] or Goal Attainment Scale (GAS) [45].

### 4.4. Implications for Clinical Practice

According to the results of this study, when providing occupational therapy interventions to patients with profiles similar to those outlined in our study, the priming effect induced by mirror visual feedback could be used to enhance sensorimotor performance after stroke. Furthermore, providing mirror visual feedback may be a better option if improved temperature sense or quality of life is the goal of treatment. Use of mirror visual feedback on bilateral task practice may increase the amount of use and quality of movement of the affected upper extremity.

## 5. Conclusions

This comparative study sheds some light on the priming effects of mirror visual feedback on improvements after stroke. Having mirror visual feedback on bilateral task practice had a better effect on the recovery of the temperature sense and quality of life. To effectively achieve treatment goals for bilateral task practice in sensorimotor rehabilitation, providing mirror visual feedback may be a better option if improvement of stroke-related quality of life is the goal of treatment. Our study also presents mirror visual feedback as a better option for the improvement of sensory function. These findings may be helpful in planning individually tailored rehabilitation therapies involving the bilateral practice approach.

## Figures and Tables

**Table 1:** Demographics and baseline clinical characteristics.

Characteristics	Mirror therapy (*n* = 12)	Bilateral arm training (*n* = 11)
Age (y)	50.72 (10.75)	58.77 (8.91)
Sex		
Male	7 (58.33%)	6 (54.55%)
Female	5 (41.67%)	5 (45.45%)
Side of lesion		
Left	5 (41.67%)	5 (45.45%)
Right	7 (58.33%)	6 (54.55%)
Type of stroke		
Ischemia	6 (50.00%)	6 (54.55%)
Hemorrhage	6 (50.00%)	5 (45.45%)
Months from stroke onset	57.92 (29.92)	47.64 (33.9)
Fugl-Meyer assessment—upper extremity	33.42 (7.48)	33 (9.74)
National Institutes of Health Stroke Scale	4.25 (2.53)	4.91 (3.51)
Education (y)	10.38 (5.13)	10.45 (3.11)

Note: data are the mean (standard deviation), median (interquartile range), or *n* (%).

**Table 2:** Descriptive and inferential statistics for outcome measures.

	Pretest scores		Posttest scores		Estimated between-group difference^‡^	*p*	*d* (95% CI)^§^
	Mirror therapy	Bilateral arm training	Mirror therapy	Bilateral arm training	Mean/median differences (95% CI)		
Fugl-Meyer Assessment—upper extremity	*n* = 12	*n* = 11	*n* = 12	*n* = 11			
Proximal	28.33 (5.03)	26.27 (6.26)	29.42 (5.18)	28.09 (5.61)	−0.74 (−2.26-0.78)	0.83	−0.40 (−1.22-0.44)
Distal	5.08 (3.53)	6.73 (5.06)	6.75 (3.39)	8.18 (5.12)	0.22 (−1.24-1.68)	0.39	0.12 (−0.70-0.94)
Total	33.42 (7.48)	33 (9.74)	36.17 (8.01)	36.27 (9.57)	−0.52 (−2.83-1.79)	0.67	−0.19 (−1.00-0.64)
Revised Nottingham Sensory Assessment^†^	*n* = 9	*n* = 6	*n* = 9	*n* = 6			
Light touch	4 (0-8)	8 (6-8)	7 (0-8)	8 (7-8)	0 (−0.42-1.42)	0.37	0.18 (−0.86-1.22)
Temperature	2 (0-4)	6 (0-8)	4 (1-7)	5.5 (0-7)	0 (0.14-2.74)	0.05	1.00 (−0.09-2.09)
Pinprick	8 (2-8)	8 (7-8)	8 (3-8)	8 (8-8)	0 (−2.31-1.75)	0.50	0.00 (−1.03-1.03)
Pressure	8 (2-8)	8 (8-8)	8 (0-8)	8 (8-8)	0 (−0.62-0.40)	0.50	0.00 (−1.03-1.03)
Localization	0 (0-5)	5 (0-7)	3 (0-4)	8 (4-8)	−1.5 (−3.14-0.14)	0.95	−1.05 (−2.15-0.05)
Bilateral simultaneous touch	7 (0-8)	8 (6-8)	4 (0-8)	8 (8-8)	0 (−1.72-0.62)	0.68	−0.26 (−1.30-0.78)
Tactile total scale	27 (6-41)	44 (27-46)	34 (11-41)	45.5 (35-47)	0 (−4.28-3.28)	0.70	−0.29 (−1.33-0.75)
	*n* = 12	*n* = 11	*n* = 12	*n* = 11			
Chedoke Arm and Hand Activity Inventory	41.42 (7.05)	42.82 (11.63)	46.58 (9.39)	50.27 (14.93)	−2.28 (−6.59-2.03)	0.85	−0.44 (−1.25-0.40)
Motor Activity Log							
Amount of use	0.58 (0.27)	0.84 (0.56)	1.37 (0.7)	1.33 (0.8)	0.30 (−0.09-0.69)	0.08	0.62 (−0.24-1.44)
Quality of movement	0.44 (0.24)	0.67 (0.6)	1.18 (0.66)	1.17 (0.73)	0.24 (−0.14-0.62)	0.12	0.50 (−0.35-1.31)
Stroke Impact Scale overall	65.46 (6.87)	64.46 (20.53)	71.38 (9.44)	64.56 (17.4)	5.82 (0.40-11.24)	0.02	0.89 (0.003-1.71)

Note: data are the mean (standard deviation) or median (interquartile range). CI = confidence interval. ^†^Only those participants who scored less than 48 points at pretest, indicating sensation impairments, were included in the data analysis. ^‡^Mean/median difference was subtracted mean/median change score of bilateral arm training group from the one of mirror therapy group. ^§^*d* is Cohen's *d* for the independent *t*-test or Mann–Whitney *U* test.

## Data Availability

The demographic and clinical data collected to support the findings of this study were approved by the Human Research Committee of the China Medical University Hospital for protecting patient privacy. The data used to support the findings of this study are available from the corresponding author upon request (kehchunglin@ntu.edu.tw).
